# Snoring, Inflammatory Markers, Adipokines and Metabolic Syndrome in Apparently Healthy Chinese

**DOI:** 10.1371/journal.pone.0027515

**Published:** 2011-11-16

**Authors:** Liang Sun, An Pan, Zhijie Yu, Huaixing Li, Aizhen Shi, Danxia Yu, Geng Zhang, Geng Zong, Yong Liu, Xu Lin

**Affiliations:** 1 Key Laboratory of Nutrition and Metabolism, Institute for Nutritional Sciences, Shanghai Institutes for Biological Sciences, Graduate School of the Chinese Academy of Sciences, Shanghai, China; 2 Department of Nutrition, Harvard School of Public Health, Boston, Massachusetts, United States of America; 3 SIBS-Novo Nordisk Translational Research Centre for PreDiabetes, Shanghai Institutes for Biological Sciences, Chinese Academy of Sciences, Shanghai, China; 4 Shanghai Municipal Center for Disease Control and Prevention, Shanghai, China; University of Hong Kong, China

## Abstract

**Objective:**

Chronic low-grade inflammation and adipokines dysregulation are linked to mechanisms underscoring the pathogenesis of obesity-related metabolic disorders. Little is known about roles of these cytokines on the association between snoring and metabolic syndrome (MetS). We aimed to investigate whether a cluster of cytokines are related to snoring frequency and its association with MetS in apparently healthy Chinese.

**Methods:**

Current analyses used a population-based sample including 1059 Shanghai residents aged 35–54 years. Self-reported snoring frequency was classified as never, occasionally and regularly. Fasting plasma glucose, lipid profile, insulin, C-reactive protein, interleukin-6, interleukin-18, lipopolysaccharide binding protein, high-molecular-weight adiponectin and leptin were measured. MetS was defined by the updated National Cholesterol Education Program Adult Treatment Panel III criteria for Asian-Americans.

**Results:**

Overweight/obese subjects had significantly higher prevalence of regular snorers than their normal-weight counterparts (34.8% vs. 11.5%, *P*<0.001). Regular snoring was associated with unfavorable profile of inflammatory markers and adipokines. However, those associations were abolished after adjustment for body mass index (BMI) or waist circumference. The MetS risk (multivariate-adjusted odds ratio 5.41, 95% confidence interval 3.72–7.88) was substantially higher in regular snorers compared with non-snorers. Controlling for BMI remarkably attenuated the association (2.03, 1.26–3.26), while adjusting for inflammatory markers and adipokines showed little effects.

**Conclusion:**

Frequent snoring was associated with an elevated MetS risk independent of lifestyle factors, adiposity, inflammatory markers and adipokines in apparently healthy Chinese. Whether snoring pattern is an economic and no-invasive indicator for screening high-risk persons needs to be addressed prospectively.

## Introduction

Habitual snoring, a manifestation of sleep-disordered breathing (SDB), has been suggested to be associated with various cardio-metabolic disorders, such as insulin resistance, hypertension, metabolic syndrome (MetS), type 2 diabetes, and cardiovascular diseases (CVD) by several epidemiological studies [1]–[7]. Although specific mechanism(s) has yet to be established, compelling evidence showed that chronic low-grade inflammation and adipokines dysregulation could mediate unfavorable effects of obesity on the pathogenesis of metabolic diseases. Previously, circulating adiponectin was reported to be associated with frequent snoring among women with type 2 diabetes, which postulated a potential mechanism explaining the relationship between snoring and cardio-metabolic disorders [8]. However, the associations of SDB/snoring with inflammatory and adipose cytokines like C-reactive protein (CRP), leptin and adiponectin were often inconsistent when taking general or central obesity into account [8]–[14].

Compared with Caucasians, Asian populations, including Chinese, tend to have higher abdominal and visceral adiposity at a given level of body mass index (BMI) [15], namely ‘metabolically obese’ phenomenon [16]. They are also thought to have higher susceptibility for metabolic diseases like type 2 diabetes than Caucasians even with lower BMI [17]. However, it is unknown whether these unique obesity phenotypes have any impact on the relationship between snoring and metabolic risk. Although excess fat mass, high levels of CRP, interleukin-18 (IL-18), leptin and low adiponectin concentration were associated with higher MetS risk in our and other populations [18]–[22], it remains to be elucidated whether the association between snoring frequency and MetS is independent or mediated through traditional risk factors, and/or unfavorable profile of inflammatory and adipose cytokines.

In light of these controversies and gaps in available literatures, we aimed to systematically investigate: 1) whether snoring frequency was related with various inflammatory markers (CRP, IL-6, IL-18 and lipopolysaccharide binding protein [LBP]) and adipokines (high-molecular-weight [HMW]-adiponectin and leptin); 2) whether snoring frequency was associated with MetS and to what extent that the association could be explained by lifestyle factors, adiposity status, inflammatory markers and adipokines in apparently healthy Chinese men and women.

## Methods

### Ethics Statement

The study was approved by the Institutional Review Board of the Institute for Nutritional Sciences and written informed consent was obtained from each participants.

### Study population

This study included a population-based case-control sample of non-institutionalized residents aged 35 to 54 years in Shanghai, China. Two urban districts (Luwan and Zhabei) were chosen to represent people from high to low socioeconomic status in urban Shanghai. Participants were enrolled through their response to an advertisement. Five hundred pairs of age and sex matched cases (overweight/obesity, BMI ≥24.0 kg/m^2^) and controls (normal-weight, 18≤BMI<24.0 kg/m^2^) were planned to be recruited. Eligible candidates were adult residents who had lived in Shanghai for at least 10 years. Exclusion criteria included 1) diarrhea for 3 consecutive days within previous 3 months; 2) heavy alcohol consumption (≥40 g/day ethanol for men and ≥20 g/day for women); 3) physician-diagnosed diabetes (or on oral anti-diabetic agents or insulin), cancer, coronary heart disease, myocardial infarction, stroke, severe kidney or liver diseases; 4) infectious diseases including tuberculosis, AIDS and hepatitis; 5) severe psychological disorders or physical disabilities; 6) antibiotics used for 3 consecutive days within previous 3 months; 7) gastrointestinal surgery within 1 year; or 8) women during pregnancy or lactation. A total of 1059 (559 overweight/obese and 500 normal-weight) subjects were successfully recruited. Information on demographic variables, health status and behaviors was obtained using a standardized questionnaire [23]. Following a home interview of the questionnaires, all participants were asked to fast overnight before having a physical examination. Body weight, height, waist circumference and blood pressure were measured using a standardized protocol [23].

Individuals without data of snoring frequency (56, 5.3%) were excluded and the final analyses included 1003 participants (392 men and 611 women), in which 534 were normal-weight and 469 were overweight/obese subjects.

### Laboratory methods

Fasting peripheral venous EDTA blood samples were collected and centrifuged at 4°C, 3000 rpm for 15 min. After being frozen, the samples were shipped in dry ice to the Institute for Nutritional Sciences and stored at −80°C until analyses. The measurements of total cholesterol, high-density lipoprotein (HDL) cholesterol, low-density lipoprotein (LDL) cholesterol, triglycerides, glucose, glycohaemoglobin (HbA1c), insulin, CRP, IL-6, IL-18, LBP, HMW-adiponectin and leptin were described previously [20], [23]. The insulin resistance index (homeostatic model assessment of insulin resistance [HOMA-IR]) was calculated according to updated homeostasis model assessment methods (http://www.dtu.ox.ac.uk/).

### Assessment of sleep, snoring and related factors

Snoring frequency was obtained from the responses to the question “Do you snore during the last month?” with 3 levels (regularly, occasionally or never). Subjective sleep quality during the last month was recorded in 3 categories (very well, fair or poor). Sleep duration was measured by self-reported average total hours of daily sleep during the previous month, including both night and nap hours of sleep and was categorized as <7.0, 7.0 to 8.9 and ≥9 hours per day, respectively.

Current smoking status was defined as never, former and current smoker. Current alcohol drinking was defined as a binary variable (yes, no). Physical activity data was collected by International Physical Activity Questionnaire (short last 7-day format, http://www.ipaq.ki.se/scoring.pdf), and level for each individual was calculated as a sum of metabolic equivalent (MET)-minute/week score and then classified as a binary variable determined by below or above the sex-specific total MET median. Educational attainment was categorized into 3 groups (0 to 9, 10 to 12 and ≥13 years of education). Family history of chronic diseases was positive if a parent or sibling was reported to have coronary heart disease, stroke or type 2 diabetes. Marital status was classified as with or without spouse/partner. Total household annual income was categorized into 3 levels (<20000, 20000 to 79999 and ≥80000 RMB).

Self-rated health status was initially recorded in 5 levels (very good, good, fair, poor and very poor) and then combined into two categories: good (very good and good) and poor (fair, poor and very poor). Center for Epidemiologic Studies Short Depression Scale (10-item CES-D scale) was used to measure the presence of clinically relevant depressive symptoms with the cut-off point of 10 [24].

### Definition of metabolic syndrome

MetS was defined according to the updated National Cholesterol Education Program Adult Treatment Panel III criteria for Asian-Americans [25], which includes at least 3 of the following components: 1) waist circumferences ≥90 cm in men or ≥80 cm in women; 2) triglycerides ≥1.7 mmol/L; 3) HDL cholesterol <1.03 mmol/L in men or <1.30 mmol/L in women; 4) blood pressure ≥130/85 mmHg, or current use of anti-hypertensive medications; and 5) fasting plasma glucose ≥5.6 mmol/L.

### Statistical analyses

Analysis of covariance for continuous variables and logistic regression models for categorical variables were applied for the characteristics comparison across snoring frequency (never, occasionally and regularly). Linear trends among cardiometabolic biomarkers across snoring frequency were analyzed by simple and multiple linear regression models. Log-transformations were performed for triglycerides, insulin, HOMA-IR, CRP, IL-6, IL-18, LBP, HMW-adiponectin and leptin to approximate normality. Multivariate logistic regression models were applied to estimate the odds ratio (OR) for MetS and its components. Adjusted potential confounders included age, sex, lifestyle factors, education level, family history of chronic diseases, marital status, annual income, self-rated health status, depressive symptoms, sleep quality, sleep duration and BMI. Inflammatory factors (CRP, IL-6, IL-18 and LBP) and adipokines (HMW-adiponectin and leptin) were further included to test whether the association was explained by these biomarkers. The ORs for MetS were also calculated according to joint classification of snoring frequency and sex, age groups (35–44, and 45–54 yrs old), obesity status (normal weight, and overweight/obesity), or cytokine index. The inflammatory index and adipokine index were computed as follows: (CRP *z* score + IL-6 *z* score + LBP *z* score + IL-18 *z* score)/4 and (adiponectin *z* score * (−1) + leptin *z* score)/2. Data management and statistical analyses were performed using Stata 9.2 (Stata, College Station, TX). Statistical tests were two-sided and *P* value<0.05 was considered statistically significant.

## Results

### General characteristics

The prevalence of regular snoring was significantly higher in overweight/obese participants than their normal-weight counterparts after controlling for age and sex (34.8% vs. 11.5%, *P*<0.001). Compared with none-snorers, snorers were more likely to be older, male, alcohol drinker, and they also have higher prevalence of MetS (all *P*<0.05; **Table 1**) and lower percentage of self-rated good health status (*P*  =  0.006). Meanwhile, regular snorers also exhibited higher levels of BMI, waist circumference and blood pressure (all *P*<0.001).

**Table 1:** Characteristics of participants across snoring frequency (n = 1003).

	Never	Occasionally	Regularly	*P* value
N	396	367	240	–
Age (yrs)^a^	45.4 (5.4)	46.5 (5.3)	46.1 (5.6)	0.020
Men (n, %)^a^	109 (27.5)	149 (40.6)	134 (55.8)	<0.001
Low physical activity (n, %)	192 (48.5)	188 (51.2)	121 (50.4)	0.732
Education level (n, %)				0.792
0∼9 yrs	98 (24.8)	99 (27.0)	75 (31.3)	
10∼12 yrs	222 (56.1)	199 (54.2)	111 (46.3)	
>12 yrs	76 (19.2)	69 (18.8)	54 (22.5)	
Current smoker (yes, n, %)	75 (18.9)	95 (25.9)	99 (41.3)	0.127
Alcohol drinker (yes, n, %)	109 (27.5)	133 (36.2)	123 (51.3)	0.005
Family history of chronic diseases (n, %)	152 (38.4)	141 (38.4)	110 (45.8)	0.076
Metabolic syndrome (n, %)	108 (27.3)	163 (44.4)	159 (66.3)	<0.001
Current marriage (n, %)	360 (90.9)	346 (94.3)	222 (92.5)	0.145
Annual income (yuan) (n = 901)				0.858
<20000	84 (22.7)	69 (21.0)	43 (21.3)	
20000–79999	236 (63.8)	221 (67.2)	138 (68.3)	
≥80000	50 (13.5)	39 (11.9)	21 (10.4)	
Self-rated good health status (n, %)	201 (50.8)	169 (46.1)	98 (40.8)	0.006
Depressive symptoms (n, %)	16 (4.0)	23 (6.3)	15 (6.3)	0.180
Sleeping pills intake (n, %)	4 (1.0)	0 (0.0)	1 (0.4)	0.523
Sleep quality (n, %) (n = 998)				0.127
Well	190 (48.5)	179 (48.9)	141 (58.8)	
Common	172 (43.9)	165 (45.1)	75 (31.3)	
Poor	30 (7.7)	22 (6.0)	24 (10.0)	
Sleep duration (n, %)				0.239
<7.0 hrs/day	80 (20.2)	63 (17.2)	56 (23.3)	
7.0–8.9 hrs/day	276 (69.7)	261 (71.1)	147 (61.3)	
≥9.0 hrs/day	40 (10.1)	43 (11.7)	37 (15.4)	
BMI (kg/m^2^)	23.3 (3.7)	24.7 (3.8)	27.0 (4.2)	<0.001
Waist circumference (cm)	80.5 (9.9)	85.5 (10.2)	92.5 (11.5)	<0.001
Systolic Blood Pressure (mmHg)	120.9 (16.8)	125.3 (17.5)	132.0 (17.8)	<0.001
Diastolic Blood Pressure (mmHg)	76.4 (11.1)	79.9 (10.9)	85.1 (12.0)	<0.001

*P* value was calculated after adjustment for age and sex. Data are arithmetic mean (SD).

a Data not adjusted for itself.

Percentages may not sum to 100 because of rounding.

Abbreviations: BMI  =  body mass index.

### Cardiometabolic biomarkers across snoring frequency

In simple linear regression models, more frequent snoring was significantly associated with higher values of insulin, HOMA-IR, triglycerides, leptin and inflammatory markers (CRP, IL-6, IL-18 and LBP), and lower concentrations of HDL and HMW-adiponectin (all *P*<0.05; **Table S1**). Controlling for age, sex and multivariate factors did not attenuate the results. However, further adjustment for BMI or waist circumference abolished the significant associations for most cardiometabolic biomarkers, particularly adiponkines and inflammatory markers.

### Associations of snoring with metabolic syndrome

The risk for MetS increased progressively across snoring frequency (*P*<0.001 for trend; **Table 2**) and regular snorers had an OR of 5.41 (95% confidence interval [CI], 3.72–7.88) compared with none-snorers, after adjusting various covariates (**Model 1**). Similar trends were also observed for the MetS components except for hyperglycemia. The ORs of MetS and its components (central obesity and hypertriglyceridemia) were attenuated but remained significant by additionally controlling for BMI (**Model 2**), and the OR for MetS was 2.03 (95% CI, 1.26–3.26) in regular snorers compared with none-snorers; while strength of the associations were not substantially altered by further adjusting for inflammatory markers and adipokines (OR for MetS, 1.95; 95% CI, 1.18–3.20; **Model 3**). Similar results were obtained after excluding 140 subjects with type 2 diabetes (**Table 2**).

**Table 2:** Odds ratios and 95% confidence interval for metabolic syndrome according to snoring frequency (n = 1003).

	Never	Occasionally	Regularly	*P* for trend
**Metabolic syndrome**	**108/396**	**163/367**	**159/240**	
Model 1	1	2.15 (1.57–2.94)	5.41 (3.72–7.88)	<0.001
Model 2	1	1.46 (0.98–2.18)	2.03 (1.26–3.26)	0.003
Model 3	1	1.45 (0.96–2.19)	1.95 (1.18–3.20)	0.007
**Metabolic syndrome** a	**78/347**	**124/316**	**122/200**	
Model 1	1	2.30 (1.61–3.27)	5.67 (3.74–8.58)	<0.001
Model 2	1	1.59 (1.02–2.47)	2.16 (1.29–3.64)	0.003
Model 3	1	1.47 (0.94–2.32)	1.98 (1.15–3.42)	0.012
**Central obesity**	**145/396**	**202/367**	**180/240**	
Model 1	1	2.44 (1.79–3.31)	6.98 (4.68–10.39)	<0.001
Model 2	1	1.97 (1.10–3.51)	2.63 (1.28–5.39)	0.005
Model 3	1	2.01 (1.10–3.65)	2.59 (1.23–5.48)	0.007
**Elevated blood pressure**	**123/396**	**158/367**	**142/240**	
Model 1	1	1.48 (1.09–2.02)	2.88 (2.00–4.14)	<0.001
Model 2	1	1.08 (0.77–1.51)	1.33 (0.88–2.00)	0.194
Model 3	1	1.06 (0.75–1.50)	1.26 (0.83–1.93)	0.300
**Hypertriglyceridemia**	**84/396**	**123/367**	**116/240**	
Model 1	1	1.76 (1.25–2.48)	3.09 (2.11–4.51)	<0.001
Model 2	1	1.38 (0.96–1.98)	1.61 (1.06–2.45)	0.023
Model 3	1	1.36 (0.94–1.97)	1.55 (1.01–2.38)	0.042
**Low HDL cholesterol**	**121/396**	**138/367**	**107/240**	
Model 1	1	1.51 (1.10–2.07)	2.12 (1.47–3.05)	<0.001
Model 2	1	1.23 (0.89–1.71)	1.24 (0.83–1.84)	0.248
Model 3	1	1.18 (0.84–1.66)	1.26 (0.83–1.91)	0.262
**Hyperglycemia**	**240/396**	**235/367**	**161/240**	
Model 1	1	1.15 (0.85–1.56)	1.32 (0.91–1.90)	0.133
Model 2	1	1.01 (0.74–1.38)	0.94 (0.63–1.40)	0.803
Model 3	1	1.03 (0.75–1.41)	0.93 (0.62–1.38)	0.770

a Excluding 140 participants who met the criteria for type 2 diabetes during the physical examinations;

Model 1: adjusted for age, sex, smoking, alcohol drinking, physical activity, education, family history of chronic diseases, marital status, annual income, self-rated health status, depressive symptoms, sleep quality and duration;

Model 2: model 1 plus BMI;

Model 3: model 2 plus inflammatory markers (CRP, LBP, IL-6 and IL-18) and adipokines (HMW-adiponectin and leptin).

Abbreviations: BMI  =  body mass index; CRP  =  C-reactive protein; HDL  =  high-density lipoprotein; HMW-adiponectin  =  high-molecular-weight adiponectin; IL  =  Interleukin; LBP  =  Lipopolysaccharide-binding protein.

**Figure 1:**
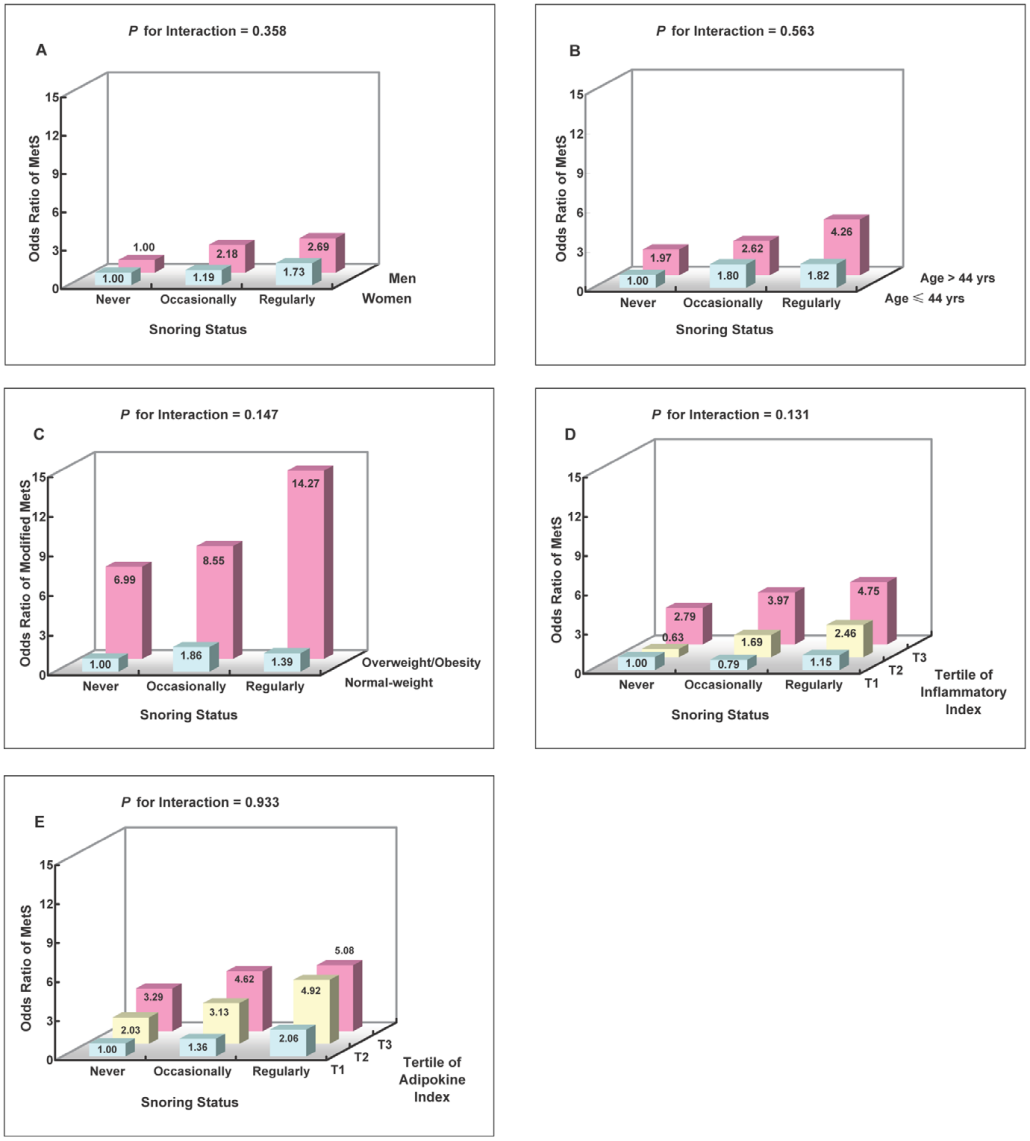
Odds ratio for metabolic syndrome according to joint classification of snoring status and sex (A), age (B), obesity status (C), inflammatory index (D) and adipokine index (E). Modified metabolic syndrome was defined as having 2 or more components of metabolic syndrome without central obesity. **A to B**: Adjusted for age (**A**), sex (**B**), smoking, alcohol drinking, physical activity, education, family history of chronic diseases, marriage status, annual income, self-rated health status, depressive symptoms, sleep quality and duration, and BMI. **C**: Adjusted for age, sex, smoking, alcohol drinking, physical activity, education, family history of chronic diseases, marriage status, annual income, self-rated health status, depressive symptoms, sleep quality and duration. **D to E**: Adjusted for covariates in C and BMI. Abbreviations: BMI  =  body mass index.

Joint classification analyses were conducted to examine whether sex, age, obesity, inflammatory index and adipokine index could modify the associations between frequent snoring and the MetS risk (**Figure 1**). No significant interactions were observed (*P*>0.05 for all interaction tests) and the association persisted in all strata.

## Discussion

Our data showed that the associations of regular snoring with unfavorable levels of inflammatory markers and adipokines were mainly explained by obese status. However, regular snoring was significantly associated with an elevated MetS risk, when potential risk factors like lifestyle factors, adiposity, inflammatory markers and adipokines were extensively controlled in apparently healthy Chinese men and women. The findings of our study provide further insights into potential mechanism(s) involved in the association between snoring status and metabolic disorders.

In consistent with the findings from a study in which snoring frequency was found to be independently associated with triglycerides, HDL and adiponectin in American diabetic women [8], we also observed that frequent snoring was significantly associated with an adverse profile of cardiometabolic biomarkers including insulin, HOMA-IR, triglycerides, HDL, adipokines (HMW-adiponectin, leptin) and inflammatory markers (CRP, IL-6, IL-18 and LBP) in an apparently healthy Chinese population. However, unlike that study, most of the associations, particularly adiponkines and inflammatory markers in our study seem to be explained by accumulated adipose status indicated by BMI or waist circumference. Indeed, controversial results have been reported in several studies when considering the influence of obesity on the associations [8]–[14]. For instance, Ulukavak and coworkers [14] observed that serum leptin was associated with apnea-hypopnea index (AHI) independent of BMI in obese Turkish SDB patients; whereas, Ip *et al* [26] indicated that body fat parameters rather than AHI were predictors for leptin levels in German SDB patients. The discrepancies among studies might be due to differences in ethnics, study design and/or severity of obesity. Since adipokines and/or proinflammatory cytokines are largely derived from adipose tissue [27], it is also unclear that to what degree the ‘metabolically obese’ phenomenon and different profile of inflammatory markers in Chinese population could influence the above associations [16], [17], [19]. Certainly, more studies are deserved in this aspect.

In the present study, BMI is showed to be the most influential factor involved in the associations of regular snoring with the MetS and its features, indicated by controlling for BMI largely attenuated the snoring-MetS association and even abolished significant associations with elevated blood pressure and low HDL cholesterol (**Table 2**). It appears that accumulating adiposity in our population served as a critical mechanistic linking between snoring and metabolic disorders. Supporting evidence also came from some of cohort and intervention studies [28]-[30]. For example, based upon the data from the Wisconsin Sleep Cohort Study, Peppard *et al* [28] discovered that weight gain was associated with an increased development and severity of SDB; whereas weight loss resulted in improvement of SDB. Notably, a strong positive association between snoring frequency and central obesity was persistent, even extensively adjusting for risk factors such as BMI, lifestyle, depressive symptoms, sleeping quality and duration, as well as multiple inflammatory cytokines and adipokines. As active endocrine organ, adipose tissue secretes a number of adipokines and promotes expression of inflammatory markers which are proposed to mediate the adverse effects of obesity on the development of metabolic diseases like type 2 diabetes and CVD [31], [32]. In addition, fat deposition in the upper airway lumen and muscle could reduce tracheal traction and lung volume, resulting in and worsening the obstruction of upper airway [33], [34], which might provide potential mechanism(s) between obesity and the pathogenesis of SDB/snoring. Taken together, findings of our study further emphasized the dominant role of obesity on the relationship between snoring and the risk of MetS when potential confounders were extensively controlled.

Our study provided more supporting evidence that frequent snoring was a strong and independent risk factor for MetS among Chinese, regardless the fact that controlling for BMI remarkably reduced the association (**Table 2**). The independent role of snoring status was also suggested by the observations that no interactions were detected between snoring with overweight/obesity, inflammatory markers or adipokines in the joint classification analyses (**Figure 1A to 1C**). Therefore, our findings implicates that frequent snoring might attribute to metabolic abnormalities *via* mechanism(s) beyond obesity and related inflammation and adipokine dysregulation. Although specific mechanism(s) is not fully understood, existing literatures suggested that SDB induced hypoxia and hypercapnia might stimulate sympathetic nervous activity [35] and generate more circulating catecholamine and cortisol [36], [37], which consequently increase insulin resistance [38]. Furthermore, hypoxia is also believed as an atherogenic factor which might increase the risk for future cardio-metabolic disturbance [39]. Previously, habitual snoring was reported to add prognostic value for type 2 diabetes and CVD independent of obesity by some of prospective studies [4]–[6]. Meanwhile, the findings from our study also supported that frequent snoring could provide additional information over putative risk biomarkers such as inflammatory markers and adipokines for MetS in apparently healthy Chinese. Collectively, self-reported snoring might be useful as a low-cost and no-invasive indicator in screening persons with higher cardio-metabolic risk, especially in developing countries.

To the best of our knowledge, this is the first study to thoroughly examine how obese status, multiple inflammatory markers and adipokines are related to snoring frequency and its association with MetS risk in the Chinese population. Our study has provided further insights in understanding the mechanism(s) linking snoring to cardio-metabolic disorders. Admittedly, there are limitations for the study. Firstly, the information of snoring habits in our study was obtained by questionnaires, a commonly used subjective method, which might introduce recall bias. However, previous studies have suggested the self-reported snoring to be a reliable measurement in epidemiology studies [40], [41]. Secondly, we did not collect information about chronic obstructive pulmonary disease (COPD), which may overlap with SDB in pathophysiology and cardio-metabolic outcomes [42]. However, existing data showed that the prevalence of COPD in urban Shanghai was 3.9% among subjects aged 40 years and older [43]. Moreover, the COPD and obstructive sleep apnea syndrome only coexist in 1% adult males [42]. Therefore, the snoring-MetS association was unlikely to be altered substantially by taking COPD into account. Moreover, the cross-sectional design could not allow establishing the causal relation. The case-control nature of the sample might limit our findings to be generalized in general populations. Certainly, future studies are needed to confirm our results prospectively in different populations.

Our study indicates that snoring frequency is significantly associated with MetS, independent of obesity, inflammatory factors and adipokines in apparently healthy Chinese. Whether snoring status could serve as an economic and no-invasive indicator for high risk individuals need to be determined prospectively.

## Supporting Information

Table S1 **Crude and adjusted measure of cardiometabolic biomarkers by snoring frequency (n = 1003).**
(DOC)
