# Supplementary material for: Snoring, Inflammatory Markers, Adipokines and Metabolic Syndrome in Apparently Healthy Chinese
Source: PLoS One. 2011 Nov 16;6(11):e27515. doi: 10.1371/journal.pone.0027515 (PMC3217970; doi:10.1371/journal.pone.0027515)
Supplement: Table S1 — Crude and adjusted measure of cardiometabolic biomarkers by snoring frequency (n = 1003). (DOC) [file pone.0027515.s001.doc]

**Table S1 Crude and adjusted measure of cardiometabolic biomarkers by snoring frequency (n=1003).**

|  | **Never** | **Occasionally** | **Regularly** | ***P* value for liner trend** |
| --- | --- | --- | --- | --- |
| **Glucose (mmol/l)** | 5.99 (0.07) | 6.11 (0.07) | 6.17 (0.09) | 0.099 |
| Model 1 | 6.26 (0.23) | 6.36 (0.23) | 6.43 (0.24) | 0.143 |
| Model 2 | 6.34 (0.22) | 6.33 (0.23) | 6.23 (0.23) | 0.428 |
| Model 3 | 6.33 (0.22) | 6.31 (0.23) | 6.20 (0.23) | 0.335 |
| **HbA1c (%)** | 5.66 (0.04) | 5.67 (0.04) | 5.77 (0.05) | 0.095 |
| Model 1 | 5.71 (0.12) | 5.71 (0.12) | 5.83 (0.12) | 0.079 |
| Model 2 | 5.75 (0.12) | 5.70 (0.12) | 5.74 (0.12) | 0.782 |
| Model 3 | 5.74 (0.12) | 5.70 (0.12) | 5.74 (0.12) | 0.885 |
| **Insulin (μU/ml)** ^a^ | 8.2 (7.8-8.7) | 9.4 (8.9-9.9) | 11.0 (10.3-11.8) | <0.001 |
| Model 1 | 8.1 (6.8-9.6) | 9.4 (7.8-11.2) | 11.3 (9.4-13.5) | <0.001 |
| Model 2 | 8.7 (7.4-10.2) | 9.2 (7.8-10.8) | 9.6 (8.1-11.3) | 0.027 |
| Model 3 | 8.6 (7.3-10.0) | 9.0 (7.7-10.6) | 9.3 (7.9-10.9) | 0.069 |
| **HOMA-IR** ^a^ | 0.96 (0.91-1.01) | 1.09 (1.04-1.16) | 1.28 (1.20-1.37) | <0.001 |
| Model 1 | 0.95 (0.80-1.13) | 1.10 (0.92-1.32) | 1.32 (1.10-1.59) | <0.001 |
| Model 2 | 1.02 (0.87-1.20) | 1.08 (0.92-1.27) | 1.12 (0.95-1.32) | 0.037 |
| Model 3 | 1.01 (0.86-1.18) | 1.06 (0.90-1.24) | 1.09 (0.92-1.28) | 0.091 |
| **Total cholesterol (mmol/l)** | 5.23 (0.06) | 5.27 (0.06) | 5.30 (0.08) | 0.484 |
| Model 1 | 5.08 (0.19) | 5.07 (0.19) | 5.16 (0.20) | 0.476 |
| Model 2 | 5.12 (0.19) | 5.06 (0.19) | 5.07 (0.20) | 0.605 |
| Model 3 | 5.11 (0.19) | 5.05 (0.19) | 5.04 (0.20) | 0.455 |
| **LDL cholesterol (mmol/l)** | 3.23 (0.05) | 3.32 (0.05) | 3.38 (0.06) | 0.053 |
| Model 1 | 3.11 (0.16) | 3.17 (0.16) | 3.28 (0.16) | 0.055 |
| Model 2 | 3.16 (0.16) | 3.16 (0.16) | 3.16 (0.16) | 0.993 |
| Model 3 | 3.16 (0.16) | 3.14 (0.16) | 3.13 (0.16) | 0.736 |
| **HDL cholesterol (mmol/l)** | 1.48 (0.02) | 1.34 (0.02) | 1.22 (0.03) | <0.001 |
| Model 1 | 1.42 (0.06) | 1.29 (0.06) | 1.20 (0.07) | <0.001 |
| Model 2 | 1.38 (0.06) | 1.30 (0.06) | 1.28 (0.06) | 0.002 |
| Model 3 | 1.38 (0.06) | 1.31 (0.06) | 1.30 (0.06) | 0.006 |
| **Triglycerides (mmol/l)** ^a^ | 1.10 (1.04-1.17) | 1.34 (1.27-1.43) | 1.63 (1.51-1.76) | <0.001 |
| Model 1 | 1.22 (1.01-1.47) | 1.43 (1.18-1.73) | 1.66 (1.37-2.02) | <0.001 |
| Model 2 | 1.30 (1.09-1.55) | 1.40 (1.17-1.67) | 1.42 (1.19-1.71) | 0.052 |
| Model 3 | 1.30 (1.09-1.54) | 1.37 (1.15-1.63) | 1.38 (1.15-1.64) | 0.170 |
| **CRP (mg/l)** ^a^ | 0.79 (0.72-0.86) | 0.92 (0.83-1.01) | 1.34 (1.19-1.51) | <0.001 |
| Model 1 | 1.09 (0.80-1.49) | 1.26 (0.92-1.73) | 1.85 (1.34-2.55) | <0.001 |
| Model 2 | 1.25 (0.94-1.65) | 1.21 (0.91-1.61) | 1.35 (1.01-1.81) | 0.429 |
| Model 3 | 1.22 (0.92-1.61) | 1.18 (0.88-1.56) | 1.31 (0.98-1.75) | 0.525 |
| **IL-6 (pg/ml)** ^a^ | 1.32 (1.24-1.40) | 1.46 (1.37-1.56) | 1.58 (1.46-1.71) | <0.001 |
| Model 1 | 1.53 (1.25-1.87) | 1.69 (1.38-2.07) | 1.79 (1.45-2.20) | 0.002 |
| Model 2 | 1.63 (1.34-1.97) | 1.66 (1.37-2.01) | 1.56 (1.28-1.91) | 0.569 |
| Model 3 | 1.61 (1.33-1.95) | 1.64 (1.35-1.99) | 1.54 (1.26-1.87) | 0.473 |
| **IL-18 (pg/ml)** ^a^ | 215.5 (207.4-223.9) | 230.1 (221.2-239.4) | 241.5 (229.9-253.6) | <0.001 |
| Model 1 | 223.4 (197.6-252.7) | 232.6 (205.2-263.6) | 239.1 (210.6-271.5) | 0.031 |
| Model 2 | 228.4 (202.4-257.9) | 231.2 (204.4-261.6) | 227.4 (200.4-258.0) | 0.984 |
| Model 3 | 227.6 (201.7-256.9) | 230.2 (203.5-260.3) | 226.3 (199.4-256.8) | 0.942 |
| **LBP (µg/ml)** ^a^ | 13.5 (11.9-15.2) | 17.3 (15.2-19.5) | 25.2 (21.6-29.4) | <0.001 |
| Model 1 | 20.1 (13.6-29.9) | 25.7 (17.2-38.5) | 37.7 (25.1-56.9) | <0.001 |
| Model 2 | 23.8 (16.6-34.0) | 24.5 (17.0-35.3) | 25.6 (17.7-37.2) | 0.501 |
| Model 3 | 23.2 (16.2-33.1) | 23.6 (16.4-33.9) | 24.6 (17.0-35.6) | 0.601 |
| **HMW-Adiponectin (µg/ml)** ^a^ | 2.84 (2.59-3.12) | 2.37 (2.16-2.61) | 1.88 (1.67-2.11) | <0.001 |
| Model 1 | 2.01 (1.50-2.70) | 1.73 (1.29-2.34) | 1.48 (1.09-2.01) | <0.001 |
| Model 2 | 1.86 (1.40-2.47) | 1.77 (1.33-2.37) | 1.77 (1.31-2.38) | 0.477 |
| Model 3 | 1.87 (1.41-2.48) | 1.81 (1.36-2.41) | 1.84 (1.37-2.47) | 0.779 |
| **Leptin (ng/ml)** ^a^ | 5.79 (5.30-6.33) | 5.83 (5.31-6.39) | 6.95 (6.21-7.79) | 0.022 |
| Model 1 | 4.50 (3.58-5.66) | 5.34 (4.23-6.75) | 7.88 (6.22-9.99) | <0.001 |
| Model 2 | 5.20 (4.37-6.20) | 5.14 (4.30-6.14) | 5.67 (4.72-6.80) | 0.138 |
| Model 3 | 5.08 (4.28-6.03) | 4.98 (4.18-5.93) | 5.47 (4.57-6.55) | 0.210 |

Data are arithmetic mean (SE). ^a^ Data are geometric mean (95% CI).

Linear regression models were used to test *P* value for linear trends.

Model 1: Adjusted for age, sex, smoking, alcohol drinking, physical activity, education, family history of chronic diseases, marital status, annual income, self-rated health status, depressive symptoms, sleep quality and sleep duration;

Model 2: model 1 plus BMI;

Model 3: model 1 plus waist circumference.

Abbreviations: CRP = C-reactive protein; HbA1c = Glycohaemoglobin; HDL = high-density lipoprotein; HMW-adiponectin = high-molecular-weight adiponectin; HOMA-IR = homeostatic model assessment of insulin resistance; IL = Interleukin; LBP = Lipopolysaccharide-binding protein; LDL = low-density lipoprotein.
